# Dynamics of HIV-1 Rev sub-nuclear trafficking reveal new insight into the role of RNA in the regulation of Rev nucleolar accumulation

**DOI:** 10.1186/1742-4690-10-S1-P103

**Published:** 2013-09-19

**Authors:** Elena Woods, Jane Courtney, Annmarie McCartin, Alessandro Marcello, William W Hall, Virginie Gautier

**Affiliations:** 1 UCD-Centre for Research in Infectious Diseases, SMMS, University College Dublin, Dublin, Ireland; 2 Dublin Institute of Technology, Kevin St, Dublin, Ireland., Dublin, Ireland; 3 Laboratory of Molecular Virology, International Centre for Genetic Engineering and Biotechnology, Trieste, Italy

## Background

The Nucleolus is a sub-nuclear compartment controlling key cellular processes, including ribosome biogenesis, transcriptional regulation, RNA trafficking and cell cycle control. Nucleolar structure is maintained through accumulation and release of retained proteins, which are selectively immobilised by RNA-protein, and/or protein-protein interactions.

As part of their replicative strategy, viruses can target the nucleolus. Specifically, the essential HIV-1 regulatory protein, Rev, selectively hijacks the Importin β/Transportin and CRM-1 transport pathways in order to shuttle between the nucleolus and the cytoplasm, facilitating the nuclear export of singly and unspliced HIV-1 mRNA. In this context, the nucleolar trafficking of HIV-1 Rev and viral transcripts could be crucial in controlling HIV-1 gene expression & replication. However, the nature of this association remains to be investigated in living cells. To better understand the molecular determinants of Rev nucleolar accumulation, we monitored HIV-1 Rev intra-nuclear movements in real time & under distinct physiological conditions using photo-convertible Dendra2 (Evrogen), and compared it the nucleolar trafficking of HIV-1 Tat, the orchestrator of HIV-1 transcription.

## Methods

Dendra2Tat and Dendra2Rev were generated by cloning Tat & Rev into the Dendra2c expression vector (Evrogen). Photoconversion and FRAP were performed on a Spinning Disk confocal microscope (Nikon) in live HeLa cells. HIV-1 transcription in HeLa-LTR-Luc and U2OS-HIV-1 cell lines was stimulated by transfecting PCAGGS-Tat, or treatment with TNF α. For nucleolar immobilisation studies, HeLa cells were heat-shocked (42ºC for 1 hour) or acidified (DMEM pH6, 2 hours). RNA Pol I was inhibited with Actinomycin D (0.08µM) for 2 hours.

## Results

Live cell imaging studies demonstrated that Dendra2Tat and Dendra2Rev accumulate but are not sequestered in the nucleoli of HeLa cells, and that Dendra2Tat is dynamically associated (T1/2 = 14 ± 2.6s) while Dendra2Rev is relatively retained (T1/2 = 31 ± 7.19s). Interestingly, HIV-1 transcription disrupted the nucleolar retention of Dendra2Rev and triggered its export to the cytoplasm.

Conversely, heat-shock and acidification increased the retention of Dendra2Rev in the nucleolus and this was dependent on RNA Pol I transcription, suggesting that Dendra2Rev nucleolar accumulation could be selectively regulated by host nucleolar ncRNA species in response to environmental cues.

## Conclusions

Our results suggest that cellular ncRNA could be a critical factor of Rev nucleolar retention and that viral transcripts compete for Rev binding and promote its release from the nucleolus.

